# Immune responses of ducks infected with duck Tembusu virus

**DOI:** 10.3389/fmicb.2015.00425

**Published:** 2015-05-08

**Authors:** Ning Li, Yao Wang, Rong Li, Jiyuan Liu, Jinzhou Zhang, Yumei Cai, Sidang Liu, Tongjie Chai, Liangmeng Wei

**Affiliations:** ^1^College of Animal Science and Veterinary Medicine, Shandong Agricultural University, Tai’an, China; ^2^Sino-German Cooperative Research Centre for Zoonosis of Animal Origin Shandong Province, Tai’an, China; ^3^Collaborative Innovation Centre for the Origin and Control of Emerging Infectious Diseases of Taishan Medical College, Tai’an, China

**Keywords:** duck, DTMUV, host innate immune response, proinflammatory cytokines, antiviral proteins

## Abstract

Duck Tembusu virus (DTMUV) can cause serious disease in ducks, characterized by reduced egg production. Although the virus has been isolated and detection methods developed, the host immune responses to DTMUV infection are unclear. Therefore, we systematically examined the expression of immune-related genes and the viral distribution in DTMUV-infected ducks, using quantitative real-time PCR. Our results show that DTMUV replicates quickly in many tissues early in infection, with the highest viral titers in the spleen 1 day after infection. *Rig-1*, *Mda5*, and *Tlr3* are involved in the host immune response to DTMUV, and the expression of proinflammatory cytokines (*Il-1*β, *–2*, *–6*, *Cxcl8*) and antiviral proteins (*Mx*, *Oas*, etc.) *are also upregulated* early in infection. The expression of *Il-6* increased most significantly in the tissues tested. The upregulation of *Mhc-I* was observed in the brain and spleen, but the expression of *Mhc-II* was upregulated in the brain and downregulated in the spleen. The expression of the interferons was also upregulated to different degrees in the spleen but that of the brain was various. Our study suggests that DTMUV replicates rapidly in various tissues and that the host immune responses are activated early in infection. However, the overexpression of cytokines may damage the host. These results extend our understanding of the immune responses of ducks to DTMUV infection, and provide insight into the pathogenesis of DTMUV attributable to host factors.

## Introduction

Duck Tembusu virus (DTMUV) is an enveloped, positive-sense, single-stranded RNA virus, classified in the genus *Flavivirus*, which includes West Nile virus (WNV), dengue virus (DENV) and other zoonotic viruses (Tang et al., 2012). DTMUV was isolated in the major duck-producing regions of China in 2010, and can cause an acute contagious infection characterized by heavy egg drop in egg-laying and breeder ducks. It is the first flavivirus reported to cause a serious epidemic disease in ducks (Cao et al., 2011; Su et al., 2011; Yan et al., 2011). Almost all species of duck can be infected with DTMUV, including Cherry Valley ducks, Pekin ducks, and shelducks (Tang et al., 2015), as can chickens, geese, and sparrows (Liu et al., 2012; Tang et al., 2013a). Most importantly, a recent study has shown that DTMUV can infect humans (Tang et al., 2013b). The serious threat DTMUV poses to the development of the duck industry and the concerns it raises for public health mean that this virus must be taken seriously.

The innate immune response is the first line of defense protecting the host from pathogenic organisms. It is well known that pattern recognition receptors (PRRs), such as Toll-like receptors (*Tlr*) 3, 7, and 8, retinoic acid inducible gene I (*Rig-1*), and melanoma differentiation factor 5 (*Mda5*), *can identify viral molecular patterns* and trigger the activation of specific signaling pathways, leading to the transcription of proinflammatory cytokines, apoptotic responses, and the expression of type I interferons (*Ifns*; Akira et al., 2006). *Tlr3*, *Rig-1*, and *Mda5* are involved in the host response to DENV and induce the production of interleukin 8 (*Cxcl8*) and *Ifn*-α/β *in vitro* (Loo et al., 2008; Green et al., 2014). Both *Rig-1* and *Mda5* recognize WNV, upregulating the expression of type I *Ifn*, *Il-1*β and antiviral effector proteins (Fredericksen et al., 2008; Quicke and Suthar, 2013).

Because DTMUV is a newly emerging virus, most studies have focused on its isolation, genetic analysis, and the establishment of diagnostic methods (Jiang et al., 2012; Li et al., 2012a,b; Zhu et al., 2012), although preliminary investigations of the pathogenicity of DTMUV have also been reported (Li et al., 2013). However, the immune responses of ducks infected with DTMUV have not been fully explored. Therefore, to clarify the innate immune responses to DTMUV in infected ducks and the tropism of the virus, we systematically investigated the expression of immune-related genes in the duck spleen and brain, and the viral titers in various tissues of infected ducks. Our study extends our understanding of the immune responses of ducks to DTMUV infection.

## Materials and Methods

### Virus Preparation

DTMUV strain FX2010, used in this study, was a gift from Zejun Li, a researcher at the Shanghai Veterinary Research Institute, Chinese Academy of Agricultural Sciences. The virus was propagated in specific-pathogen-free embryonated chicken eggs and the titers were shown to be 10^5.2^ median tissue culture infective doses (TCID_50_)/mL in infected duck embryonic fibroblasts, calculated with the Reed and Muench method (Reed and Muench, 1938).

### Animal Experiment

One-day-old Cherry Valley ducks were purchased from a duck farm (Tai’an, Shandong) and housed in isolators until use. The ducks were confirmed to be serologically negative for DTMUV using a blocking enzyme-linked immunosorbent assay (Li et al., 2012b). All animal experiments were performed according to the guidelines of the Committee on the Ethics of Animals of Shandong and the appropriate biosecurity guidelines. At 5 days old, the ducks were randomly divided into two groups, each containing 25 animals. The ducks of one group were infected intramuscularly with 0.4 mL of 10^5.2^ TCID_50_ virus. The control group was inoculated in the same manner with 0.4 mL of sterile phosphate-buffered saline (PBS). Three live ducks, except the dead ducks, from each group were euthanized at 1, 2, 3, 4, and 5 days post infection (dpi) and their parenchymatous organs (heart, liver, spleen, lung, kidney, brain, and pancreas) were collected and stored at –70°C until viral titration and the analysis of immune-related gene expression. The remaining ducks were observed for clinical symptoms for 9 days and were euthanized with an intravenous injection of sodium pentobarbital (100 mg/kg bodyweight) at the end of the study (Pantin-Jackwood et al., 2012).

### RNA and cDNA Preparation

The collected tissues (0.1 g) were ground in liquid nitrogen and the total RNAs were extracted from the tissues with TRIzol Reagent (Takara, Dalian, China), according to the manufacturer’s instructions. The concentrations of the total RNAs were measured with an ultraviolet spectrophotometer (Shimadzu, Shimazu, Japan). A sample of each RNA (1 μg) was treated with DNase I (Thermo Scientific, Lithuania) and reverse transcribed with M-MLV reverse transcriptase (Promega, Madison, WI, USA). The synthesized cDNA was stored at –20°C until analysis.

### Quantitative Real-time PCR

The relative expression of immune-related genes was quantified after infection using previously described primers (Wei et al., 2013a, 2014). The primers for the *Ifn*-β gene were designed using the Primer 3 software, based on the published GenBank sequence (GenBank: KM035791.1). The primers for the *E* gene of DTMUV were as previously reported (Yu et al., 2012). To confirm the copy numbers of DTMUV in the affected ducks, the viral titers (log_10_) were normalized to 1 μg of total RNA. Quantitative real-time PCR was performed with the 7500 Fast Real-Time PCR System (Applied Biosystems, Carlsbad, CA, USA) using the SYBR Green PCR kit (Takara, Dalian, China). All primer pairs (Table 1) were selected according to their specificity, determined with dissociation curves. Quantitative real-time PCR was performed in a reaction volume of 20 μL, according to the manufacturer’s instructions. The PCR cycling conditions were: one cycle at 95°C for 30 s, 40 cycles of denaturation at 95°C for 5 s and extension at 60°C for 34 s, followed by a dissociation curve analysis step. To validate the assay, the purified PCR products were cloned into the pMD18-T plasmid and sequenced to confirm the proper amplification. Each sample was analyzed in triplicate.

**TABLE 1:** **Primers used in this study**.

**Primer name**	**Sequence (5′–3′)**	**Product size (bp)**	**GenBank no.**
Rig-1 F	GCTACCGCCGCTACATCGAG	224	EU363349
Rig-1 R	TGCCAGTCCTGTGTAACCTG		
Mda5 F	GCTACAGAAGATAGAAGTGTCA	120	KJ451070.1
Mda5 R	CAGGATCAGATCTGGTTCAG		
Tlr3 F	GAGTTTCACACAGGATGTTTAC	200	JQ910167
Tlr3 R	GTGAGATTTGTTCCTTGCAG		
IL-1β F	TCATCTTCTACCGCCTGGAC	149	DQ393268
Il-1β R	GTAGGTGGCGATGTTGACCT		
Il-2 F	GCCAAGAGCTGACCAACTTC	137	AF294323
Il-2 R	ATCGCCCACACTAAGAGCAT		
Il-6 F	TTCGACGAGGAGAAATGCTT	150	AB191038
Il-6 R	CCTTATCGTCGTTGCCAGAT		
Cxcl8 F	AAGTTCATCCACCCTAAATC	182	DQ393274
Cxcl8 R	GCATCAGAATTGAGCTGAGC		
Ifn-α F	TCCTCCAACACCTCTTCGAC	232	EF053034
Ifn-α R	GGGCTGTAGGTGTGGTTCTG		
Ifn-β F	AGATGGCTCCCAGCTCTACA	210	KM035791.1
Ifn-β R	AGTGGTTGAGCTGGTTGAGG		
Ifn-γF	GCTGATGGCAATCCTGTTTT	247	AJ012254
Ifn-γR	GGATTTTCAAGCCAGTCAGC		
Mx F	TGCTGTCCTTCATGACTTCG	153	GU202170.1
Mx R	GCTTTGCTGAGCCGATTAAC		
Oas F	TCTTCCTCAGCTGCTTCTCC	187	KJ126991.1
Oas R	ACTTCGATGGACTCGCTGTT		
Pkr F	AATTCCTTGCCTTTTCATTCAA	109	Unpublished
Pkr R	TTTGTTTTGTGCCATATCTTGG		
Mhc-I F	GAAGGAAGAGACTTCATTGCCTTGG	196	AB115246
Mhc-I R	CTCTCCTCTCCAGTACGTCCTTCC		
Mhc-II F	CCACCTTTACCAGCTTCGAG	229	AY905539
Mhc-II R	CCGTTCTTCATCCAGGTGAT		
DTMUV-E F	CGCTGAGATGGAGGATTATGG	225	KC990541.1
DTMUV-E R	ACTGATTGTTTGGTGGCGTG		
β-actin F	GGTATCGGCAGCAGTCTTA	160	EF667345.1
β-actin R	TTCACAGAGGCGAGTAACTT		

### Statistical Analysis

The relative expression of the target genes in the infected and control groups was calculated with the 2^–ΔΔCt^ method and expressed as the fold changes in gene expression. The housekeeping gene encoding β-*actin* (*Actb*) was used as the endogenous control against which to normalize the expression levels of the target genes. The fold changes were logarithmically transformed. All data were analyzed with Student’s *t*-test using GraphPad Prism 5 (GraphPad Software Inc., San Diego, CA, USA). Statistical significance was set at *P* < 0.05.

## Results

### Clinical Symptoms and Viral Titers in DTMUV-infected Ducks

The clinical symptoms of the affected ducks were observed at 3 dpi and the ducks showed loss of appetite and depression and were reluctant to move. At 4–6 dpi, some ducks appeared neurological signs, such as dystaxia and paralysis. In present study, four infected ducks died at 4 dpi and three died at 5 dpi. The symptoms of ducks affected with DTMUV gradually lessened and disappeared at 9 dpi.

**FIGURE 1:**
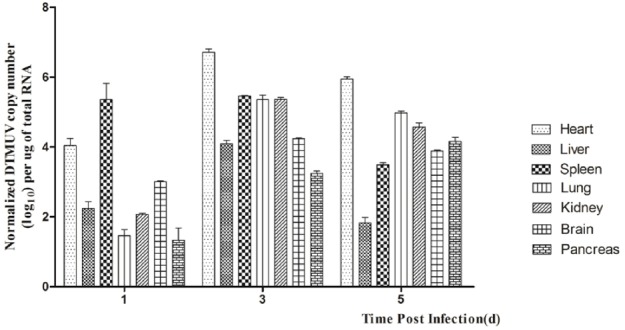
**Viral titers in DTMUV-infected ducks at 1, 2, and 3 dpi.** Data are expressed as means ± standard deviations (*n* = 3), and each sample was analyzed in triplicate.

In this study, we detected DTMUV replication in the parenchymatous organs of infected ducks in the first 5 days after infection. As shown in Figure 1, the viral titers could be detected in all the tissues tested at 1 dpi and the highest titer was observed in the spleen. In the same period, DTMUV replicated rapidly in the brain, to a high titer. The viral titers in all tissues peaked at 3 dpi, except in the spleen and pancreas. The viral titer in the heart reached 10^6.7^ copies, and those in the spleen, lung and kidney were basically identical. The viral titers in most of the tested tissues began to decline at 5 dpi, but were still highest in the heart. The viral titer in the spleen decreased dramatically at 5 dpi compared with that at 3 dpi, whereas the viral titer in the brain reached 10^3.8^ copies. No virus was detected in the control group. In summary, DTMUV replicated quickly in many organs, leading to systemic impairment.

### Expression of PRR mRNAs in DTMUV-infected Ducks

**FIGURE 2:**
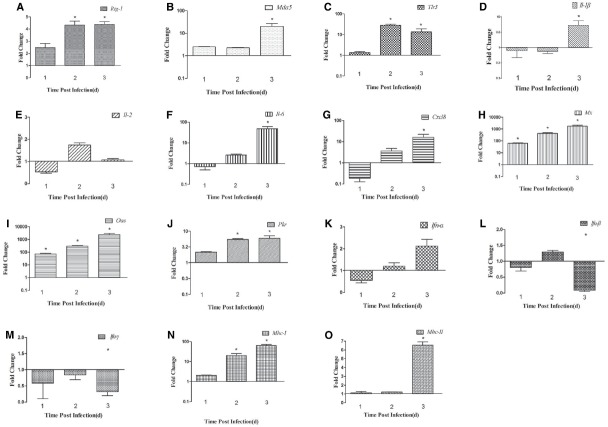
**Expression of immune-related genes in the brains of DTMUV-infected ducks. (A)** *Rig-1*, **(B)** *Mda5*, **(C)** *Tlr3*, **(D)** *Il-1*β, **(E)** *Il-2*, **(F)** *Il-6*, **(G)** *Cxcl8*, **(H)** *Mx*, **(I)** *Oas*, **(J)** *Pkr*, **(K)** *Ifn*-α, **(L)** *Ifn*-β, **(M)** *Ifn*-γ, **(N)** *Mhc-I*, and **(O)** *Mhc-II*. The Y axis represents the fold change in target gene expression in the experimental group versus that in the control group. Data are expressed as means ± standard deviations (*n* = 3). Differences were evaluated with Student’s *t*-test and were considered significant at *P < 0.05.

We detected the expression of PRRs (*Rig-1*, *Mda5*, and *Tlr3*) in the brains and spleens of ducks infected with DTMUV during the early post infection period. In the brain, the expression of *Rig-1* and *Mda5* was upregulated during the first 3 days of infection, and peaked at 2 dpi and 3 dpi, respectively (4.13-fold and 20.60-fold, respectively, *P* < 0.05; Figures 2A,B). *Tlr3* was expressed at 1 dpi (1.35-fold), peaked at 2 dpi (28.54-fold, *P* < 0.05), and remained high at 3 dpi (13.49-fold, *P* < 0.05; Figure 2C).

**FIGURE 3:**
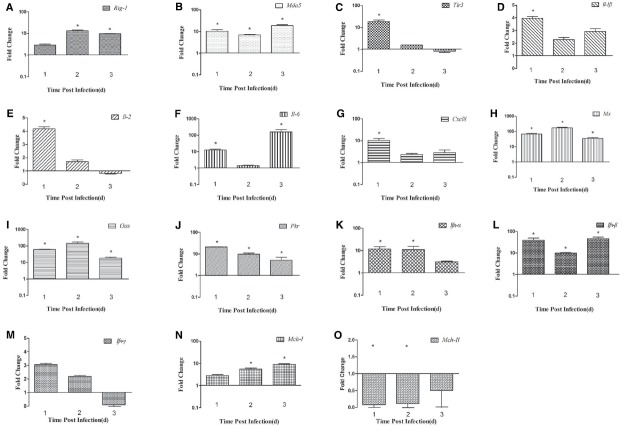
**Expression of immune-related genes in the spleens of DTMUV-infected ducks. (A)** *Rig-1*, **(B)** *Mda5*, **(C)** *Tlr3*, **(D)** *Il-1*β, **(E)** *Il-2*, **(F)** *Il-6*, **(G)** *Cxcl8*, **(H)** *Mx*, **(I)** *Oas*, **(J)** *Pkr*, **(K)** *Ifn*-α, **(L)** *Ifn*-β, **(M)** *Ifn*-γ, **(N)** *Mhc-I*, and **(O)** *Mhc-II*. The Y axis represents the fold change in target gene expression in the experimental group versus that in the control group. Data are expressed as means ± standard deviations (*n* = 3). Differences were evaluated with Student’s *t*-test and were considered significant at *P < 0.05.

In the spleen, the expression of *Rig-1* was upregulated at 1 dpi (2.89-fold) and peaked at 2 dpi (13.62-fold, *P* < 0.05), and then decreased slightly at 3 dpi (9.71-fold, *P* < 0.05; Figure 3A). *Mda5* transcripts were detected at 1 dpi (10.29-fold, *P* < 0.05), peaked at 3 dpi (18.77-fold, *P* < 0.05; Figure 3B). There was an 18.34-fold increase in *Tlr3* mRNA at 1 dpi, which then decreased significantly at 2 dpi (1.57-fold) and decreased further at 3 dpi (0.81-fold; Figure 3C). These data indicate that *Rig-1*, *Mda5*, and *Tlr3* are involved in the host immune response to DTMUV, and that the roles they play might differ with time.

### Cytokine Expression in DTMUV-infected Ducks

To determine the induction of proinflammatory cytokines in ducks infected with DTMUV, we determined the expression levels of *Il-1*β, *Il-2*, *Il-6*, *Cxcl8*, and the type I and II *Ifn* genes. In the brain, the expression of *Il-1*β was downregulated at 1 dpi and 2 dpi, but upregulated at 3 dpi (5.30-fold, *P* < 0.05; Figure 2D). *Il-2* expression was downregulated 0.53-fold at 1 dpi but upregulated 1.73-fold at 2 dpi, after which it decreased slightly at 3 dpi (1.08-fold; Figure 2E). The expression of *Il-6* and *Cxcl8* showed similar tendencies during the 3 days tested, with reduced expression (0.72-fold and 0.18-fold, respectively) at 1 dpi, which gradually increased to a peak at 3 dpi (47.78-fold and 16.05-fold, respectively, *P* < 0.05; Figures 2F,G). The patterns of type I and II *Ifn* expression differed in the brain. The expression of *Ifn*-α was downregulated at 1 dpi (0.55-fold) and gradually upregulated at 2 dpi, reaching its highest level at 3 dpi (1.96-fold; Figure 2K). The expression of *Ifn*-β was downregulated at 1 dpi, slightly upregulated at 2 dpi (1.29-fold), and downregulated again at 3 dpi (Figure 2L). The expression of *Ifn*-γ was downregulated at all time points (Figure 2M).

In the spleen, the expression of *Il-1*β and *Cxcl8* was highest at 1 dpi (3.94-fold and 10.36-fold, respectively, *P* < 0.05), and then decreased slightly in the following 2 days (Figures 3D and G). The expression of *Il-2* was upregulated at 1 dpi (4.17-fold, *P* < 0.05), decreased gradually by 2 dpi, and was downregulated at 3 dpi (0.82-fold; Figure 3E). *Il-6* mRNA expression increased constantly, peaking at 3 dpi (160.10-fold, *P* < 0.05; Figure 3F). The high expression of *Ifn*-α was maintained for 2 days (11.84-fold at 1 dpi and 11.02-fold at 2 dpi, *P* < 0.05), but decreased to 3.10-fold at 3 dpi (Figure 3K). The expression of the *Ifn*-β gene was markedly upregulated at 1 dpi and 3 dpi (38.38-fold and 46.63-fold, respectively, *P* < 0.05), but less so at 2 dpi (9.78-fold; Figure 3L). Unlike its expression in the brain, the expression of the *Ifn*-γ gene was higher than in the control at 1 dpi and 2 dpi (3.05-fold and 2.18-fold, respectively), but was downregulated at 3 dpi (0.09-fold, *P* < 0.05; Figure 3M). These results show that the expression of various cytokines is induced in DTMUV-infected ducks, and that the expression patterns of some cytokines are variable. In summary, the expression of *Il-6* was most significantly increased during the early period of DTMUV infection and the type I *Ifns* played a key role in the duck’s response to DTMUV in the same period.

### Expression of Antiviral Proteins in DTMUV-infected Ducks

Antiviral proteins are effective components of the response to viral infections, so we investigated the expression of several antiviral proteins, including MX, OAS, and PKR. The three antiviral proteins showed similar trends in the brain: the expression of all of them increased during the 3 days tested. In the brain, the expression of the *Mx* and *Oas* genes was upregulated 65.01-fold and 72.84-fold, respectively, at 1 dpi (*P* < 0.05), increased 431.61-fold and 298.52-fold, respectively, at 2 dpi (*P* < 0.05), and showed dramatic increases of 1733.20-fold and 2375.16-fold, respectively, at 3 dpi (*P* < 0.05; Figures 2H,I). Pkr expression was gradually increasing during the tested days and peaked at 3 dpi (5.91-fold, *P* < 0.05; Figure 2J). However, in the spleen, the expression of these antiviral proteins was variable. *Mx* and *Oas* expression was significantly increased at 1 dpi (67.26-fold and 60.97-fold, respectively; *P* < 0.05) and at 2 dpi (172.67-fold and 144.55-fold, respectively; *P* < 0.05), and then decreased at 3 dpi (Figures 3H,I). *Pkr* mRNA expression was significantly upregulated at 1 dpi (21.07-fold, *P* < 0.05), and gradually declined in the following 2 days (9.81-fold and 5.06-fold, respectively, *P* < 0.05; Figure 3J).

These data demonstrate that the expression of some antiviral proteins, especially MX and OAS, increased significantly in the brains and spleens of DTMUV-infected ducks, indicating that they play important roles in resisting DTMUV infection.

### Expression of MHC Class I and II Molecules in DTMUV-infected Ducks

To confirm whether MHC-I and -II molecules are involved in the host immune responses to DTMUV, we examined their expression in the first 3 days after infection. The expression of both *Mhc-I* and *-II* molecules was upregulated in the brains of the infected ducks, peaking at 3 dpi (64.08-fold and 6.26-fold, respectively, *P* < 0.05; Figures 2N,O). In the spleen, *Mhc-I* gene expression was upregulated 2.84-fold at 1 dpi and 5.55-fold at 2 dpi, peaking at 3 dpi (8.99-fold, *P* < 0.05; Figure 3N). However, the expression of *Mhc-II* molecules was downregulated in the spleen on all 3 days examined (Figure 3O). These results indicate that both MHC-I and -II molecules are involved in the duck immune responses to DTMUV.

## Discussion

It has been reported that Tembusu virus causes a disease in chickens that is characterized by encephalitis and growth retardation (Kono et al., 2000), but does not do so in ducks. However, DTMUV caused serious outbreaks of disease in ducks in 2010, involving severe economic losses (Su et al., 2011; Yan et al., 2011). This difference in the pathogenicity of Tembusu virus is determined by many factors, especially the host immune response. Here, we systematically examined the expression of immune-related genes at the mRNA level and the distribution of the virus in DTMUV-infected ducks.

The pathogenicity of DTMUV in ducks correlates directly with the level of virus in the tissues. In this study, the viral titer at 1 dpi was highest in the spleen, indicating that the spleen is the target organ of DTMUV (Jiang et al., 2012). The viral titers in all the tissues tested peaked at 3 dpi, except in the spleen and pancreas, and gradually decreased to 5 dpi (Figure 1). These results show that DTMUV replicates rapidly in the parenchymal organs, including the brain, and that DTMUV causes viremia and disrupts the blood–brain barrier in a short period of time. High levels of virus in their tissues may have been the main cause of death in some infected ducks.

The innate immune response of the host is the primary mechanism for resisting and clearing viruses during the early stage of infection. Viral genomes and replication products are sensed by key PRRs, such as *Rig-1*, *Mda5*, and Tlr3/7/8 (Pichlmair and Reis e Sousa, 2007), and both WNV and DENV-2 trigger RIG-1 and MDA5 signaling (Fredericksen et al., 2008; Green et al., 2014). However, The role of TLR3 involved in Flavivirus infection is controversial. It has recently been demonstrated that DENV activates TLR3 signaling cascades, leading to the transcription of IFN-α/β in mononuclear cells (Tsai et al., 2009). WNV inhibited the TLR3-mediated production of IL-6 and an antiviral state (Scholle and Mason, 2005; Wilson et al., 2008), and Wang et al. (2004) had proved that viral titers and neuropathology were reduced in the brain of WNV-infected TLR3-deficient mice comparing to the control (Wang et al., 2004), which suggesting TLR3-mediated inflammatory response may disrupt the blood-brain barrier and accelerate the WNV into the CNS (Fredericksen and Gale, 2006; Matsumoto et al., 2011). In our study, the expression of *Rig-1*, *Mda5*, and *Tlr3* was upregulated in the brain and spleen during the period of infection tested, although the expression of *Tlr3* was not upregulated in the spleen at 3 dpi (Figures 2C and 3C). We also found that *Tlr3* expression was significantly upregulated in the brain at 2 dpi (28.54-fold, *P* < 0.05), but decreased to 13.49-fold at 3 dpi, whereas *Mda5* was markedly increased (20.60-fold) at that time (Figures 2B,C). In the spleen, the expression of *Tlr3* increased at 1 dpi (18.34-fold, *P* < 0.05), but decreased to 1.57-fold at 2 dpi and was further downregulated at 3 dpi, whereas *Rig-1* and *Mda5* were significantly upregulated at 2 dpi and 3 dpi (13.62-fold and 18.77-fold, respectively, *P* < 0.05; Figures 3A,B). These results suggest that the different PRRs may play key roles at different times. It was recently reported that RIG-1 and MDA5 are required for the recognition of WNV: RIG-1 is considered to trigger the expression of immune-related genes early in infection, whereas MDA5 signaling occurs later (Errett et al., 2013).

The activation of PRRs induces the expression of cytokines and antiviral proteins, including IL-1β, IL-2, TNF-α, MX, and OAS, which alert the immune system to viral infection. Here, we examined several cytokines and found that the expression of *Il-1*β, *Il-2*, *Il-6*, and *Cxcl8* increased in the spleen on the days examined, with *Il-6* expression particularly elevated (160.12-fold at 3 dpi, *P* < 0.05). In the brain, all the cytokines tested were downregulated at 1 dpi, but upregulated at 3 dpi, and *Il-6* was again most strongly upregulated (47.78-fold, *P* < 0.05). A previous study suggested that *Il-6* is more robustly induced in chickens than in ducks, which may be responsible for the different symptoms observed in the two species after influenza virus infection (Liang et al., 2011). In mammals, “cytokine storms” are believed to contribute to more severe pathological lesions and higher rates of death (Chan et al., 2005). Similar results were also observed in ducks infected with the highly pathogenic avian influenza virus H5N1 (Wei et al., 2013a). In the present study, 28% of the DTMUV-infected ducks died, suggesting that the excessive expression of cytokines, such as *Il-6* and *Cxcl8*, and the rapid replication of DTMUV in various tissues may have caused the deaths of the infected ducks.

Type I IFN production is a typical innate defense against viral infection and the expression of antiviral proteins contributes to viral clearance. The expression of the most genes including the Mx, Oas have increased in brain and spleen from the mice infected with WNV, which suggesting that the gene products may be involved in the protection against WNV (Venter et al., 2005). In this study, *Ifn-α/*β expression was significantly induced in the spleen early in infection. The expression of *Mx* and *Oas* increased significantly in the brain and spleen, but failed to prevent massive viral replication and was insufficient to protect the ducks from DTMUV. A similar phenomenon has been observed in geese and chickens infected with highly pathogenic avian influenza virus H5N1 (Daviet et al., 2009; Wei et al., 2013b).

MHC molecules can activate the acquired immune response to eliminate a viral infection, and some viruses inhibit MHC-I expression. In our study, the upregulation of *Mhc-I* was observed in the brains and spleens of infected ducks (Figures 2N and 3N), which is not surprising because the upregulation of *Mhc-I* has been observed during infection with DENV and WNV. This phenomenon has only been observed in the genus *Flavivirus*, and not in the other two genera, hepatitis C virus and the pestiviruses. However, the definitive role of *Mhc-I* upregulation during *Flavivirus* infection is unclear (Lobigs et al., 2003). In the present study, the production of *Mhc-II* increased slightly in the brain, but was downregulated in the spleen throughout the experimental period (Figures 2O and 3O). *Mhc-II* is also reportedly downregulated in response to avian influenza virus infection *in vivo* and *in vitro* (Adams et al., 2009; Liang et al., 2011; Cagle et al., 2012).

In summary, DTMUV induces the upregulation of *Rig-1*, *Mda5*, and *Tlr3* expression in ducks, resulting in the activation of *Ifns* and several interferon-stimulated genes, including proinflammatory cytokines and antiviral proteins. Although various antiviral proteins and *Ifns* were induced, they did not provide adequate protection against DTMUV infection in ducks, and the excessive host immune responses, including massive *Il-6* expression, and the rapid replication of DTMUV damaged the host, leading to serious disease and even death. As far as we know, this is the first report of the immune-related gene expression in response to DTMUV infection in ducks. We have attempted to provide a comprehensive picture of the duck immune responses to DTMUV infection. Our results provide useful information concerning the relationship between DTMUV and the host immune response, and insight into the pathogenesis of DTMUV attributable to host factors.

### Conflict of Interest Statement

The authors declare that the research was conducted in the absence of any commercial or financial relationships that could be construed as a potential conflict of interest.
